# Auditory Neuropathy Caused by a Structural Variation in the *OTOF* Gene, Identified Using Oxford Nanopore Adaptive Sampling

**DOI:** 10.3390/genes16020116

**Published:** 2025-01-21

**Authors:** Takumi Kumai, Shin-ya Nishio, Hideaki Moteki, Akihiro Katada, Shin-ichi Usami

**Affiliations:** 1 Department of Otolaryngology-Head and Neck Surgery, Asahikawa Medical University, Asahikawa 078-8510, Japan; 2 Department of Hearing Implant Sciences, Shinshu University School of Medicine, Matsumoto 390-8621, Japan; nishio@shinshu-u.ac.jp (S.-y.N.); hideaki.moteki@gmail.com (H.M.); 3 Department of Otolaryngology, Asahikawa Red Cross Hospital, Asahikawa 070-0061, Japan; a.katada@asahikawa-rch.gr.jp

**Keywords:** *OTOF*, DFNB9, auditory neuropathy, ANSD, copy number variation, long read, next-generation sequencing, nanopore, adaptive sampling

## Abstract

Background/Objectives: The *OTOF* gene is reported to be the causative gene for non-syndromic recessive sensorineural hearing loss and auditory neuropathy spectrum disorder. About 300 variants have been reported, but there have been no reports to date on copy gain variants. Methods: We identified a copy gain variant in the *OTOF* gene through short-read next-generation sequencing analysis from one patient with auditory neuropathy. We also performed long-read next-generation sequencing analysis using the Oxford Nanopore Technologies adaptive sampling procedure. Results: The four-year-old male carried a duplication of chr2: 26,477,852 to 26,483,106 (a 5254-base duplication including exon 14 to exon 18 of the *OTOF* gene NM_001287489) and a c.5385C>A single nucleotide variant. We also confirmed that these two variants were located in the *trans* configuration based on haplotype phasing results using the long-read next-generation sequencing data. Conclusions: This is the first report of an auditory neuropathy patient with a large duplication variant in the *OTOF* gene. The identified variants were novel, but based on the clinical phenotype of the patient, these variants seem to be the genetic cause of this patient’s phenotype. Oxford Nanopore Technologies adaptive sampling is a powerful tool for the analysis of structural variants (particularly for determining the breakpoint and direction) and haplotype phasing.

## 1. Introduction

Hearing loss (HL) is one of the most common sensory disorders, and was observed in 1.62 of 1000 newborns in our recent epidemiological survey result for 153,913 newborns [1]. In this epidemiological survey, we also analyzed the etiology of HL and revealed that 56.1% of cases of congenital-onset bilateral HL were attributable to genetic causes [1]. To date, 89 genes have been identified as causative genes for autosomal recessive non-syndromic HL (ARNSHL), which is the most common type of hereditary HL [2].

The *OTOF* gene is reported to be the causative gene for non-syndromic recessive sensorineural HL (DFNB9) [3] and is also reported as a major causative gene for auditory neuropathy spectrum disorder (ANSD) [4], which is characterized by an abnormal auditory brainstem response (ABR) or auditory steady state response (ASSR) with the normal appearance of otoacoustic emissions (OAE) [5]. Moreover, acoustic stapedial reflex could be absent. The *OTOF* gene encodes the otoferlin protein, which plays a crucial role in the function of inner hair cells (IHCs), specifically in the synaptic vesicle and neurotransmitter exocytosis from IHCs [6]. *OTOF* gene mutations give rise to the ANSD phenotype (also known as auditory synaptopathy), which presents with synaptic dysfunction between IHCs and spiral ganglion neurons [7].

*OTOF* is a relatively rare causative gene in relation to ARNSHL. The prevalence of *OTOF* gene-associated HL is reported to represent 0.8–3.2% of non-syndromic HL cases [8,9,10,11,12,13]. On the other hand, the *OTOF* gene is the most prevalent cause of ANSD, with 41–90% of pediatric cases of ANSD attributable to *OTOF* gene mutations [8,14,15,16]. Unlike “true” auditory neuropathy, *OTOF*-associated HL shows intact auditory nerve function, and generally favorable cochlear implant (CI) outcomes are observed [17,18].

Recently, *OTOF*-associated HL has received special attention as a promising candidate for gene therapy. Several companies have begun phase-1/2 clinical trials of gene therapies for *OTOF*-associated HL. In addition, a first report of these clinical trials indicated hearing improvements through gene therapy in *OTOF*-associated HL patients [19]. The main inclusion criteria for this clinical trial were children (aged 1–18 years) with severe-to-complete hearing loss as defined by the average of the auditory brainstem response (ABR) threshold (0.5, 1, 2, 4 kHz) with confirmation of the participant’s body temperature within a normal range (36–37 °C) and the presence of the pathogenic variant in both alleles of the *OTOF* gene without bilateral cochlear implants [19]. Needless to say, prior to any gene therapy, appropriate genetic diagnosis is important to select the suitable candidates for gene therapy.

Here, we report an *OTOF*-associated HL patient with a single nucleotide variant (SNV) and structural variation (a duplication including the exon 14 to exon 18 region of the *OTOF* gene NG_009937.1(NM_001287489)). This structural variation was identified by using the read depth data obtained from short-read next-generation DNA sequencing (NGS). We also performed long-read NGS analysis to determine the break points using the Oxford Nanopore Technologies adaptive sampling procedure. This is the first report to identify a large copy gain variant in an *OTOF*-associated ANSD patient. In addition, we determined the breakpoint of this large copy gain variant by using long-read NGS with the adaptive sampling procedure.

## 2. Materials and Methods

### 2.1. Subject

The patient was a four-year-old boy with auditory neuropathy spectrum disorder. He visited the Department of Otolaryngology, Asahikawa Medical University, for his HL in 2020. Detailed clinical data were collected from his medical records. Genetic analysis of this patient was conducted in 2023. This study was approved by the Shinshu University Ethical Committee (Approval number: No. 718—7 March 2022), and was conducted in accordance with the Declaration of Helsinki. Informed consent was obtained from his parents prior to enrollment in this study.

### 2.2. Short-Read Next-Generation Sequencing Analysis

In this study, we examined 63 genes previously reported to be associated with non-syndromic HL or syndromic HL. The detailed protocols for targeted enrichment and DNA sequencing are reported elsewhere [20]. In brief, amplicon libraries were prepared with the Ion AmpliSeq Custom Panel, Ion AmpliSeq Library Kit 2.0, and the Ion Xpress Barcode Adapter 1–96 Kit (Life Technologies, Carlsbad, CA, USA) according to the manufacturer’s instructions. After the amplicon libraries were prepared, we performed short-read next-generation sequencing analysis using the Ion S5 system with an Ion 540 chip according to the manufacturer’s instructions. The sequence data were mapped against the human genome sequence (build GRCh38.p5) with the Torrent Mapping Alignment Program. Subsequently, DNA variants were piled up with the Torrent Variant Caller plug-in software ver 5.16. included in the Torrent Suit (Life Technologies). After variant detection, the effects of the variants were analyzed using ANNOVAR software ver. 2020-06-08 [21]. The missense, nonsense, insertion/deletion and splicing variants were selected among the identified variants. Variants were further selected as described in our previous report [13]. The pathogenicity of the identified variants was evaluated using the American College of Medical Genetics (ACMG) standards and guidelines [22] with ClinGen HL expert panel specification [23]. Copy number variant (CNV) analysis was performed using the read depth data according to the method described in our previous report [24].

### 2.3. Long-Read Next-Generation Sequencing Analysis

Long-read NGS analysis was performed for this patient to determine the break points in the CNV identified by the short-read NGS analysis. The sequencing library was prepared using the Rapid Sequencing Kit V14 (Oxford Nanopore Technologies, Oxford, UK) according to the manufacturer’s instructions. Sequencing was performed using PromethION 2 solo with a PromethION flow cell ver. R10.4.1 (Oxford Nanopore Technologies, Oxford, UK) with the adaptive sampling option (enriched target sequencing). The target region for adaptive sampling used in this study was extracted from the reference human genome GRCh38.p5. The target sequence was set for the *OTOF* gene and the peripheral one million bases (ranging from chr2: 25,457,203 to chr2: 27,558,756). Base calling was performed in the high accuracy mode for GPU base calling on a personal computer with NVIDIA RTX A6000 ada (NVIDIA, Santa Clara, CA, USA). After sequencing, the sequence data were mapped against the human genome sequence (build GRCh38.p5) with the NGMLR ver.0.2.7 [25]. After sequence mapping, variant calling and haplotype phasing were performed using Clair3 ver.0.1-r10 with the enable_phasing option and enable_long_indel option [26]. Using the phased variant calling results and bam file, we created a haplotagged bam file and split it into separate bam files with WhatsHap ver.1.0 [27]. The mapping data were visualized with J Brows 2 ver.2.16.1 [28].

## 3. Results

### 3.1. Clinical Details of the Subject

**Figure 1:**
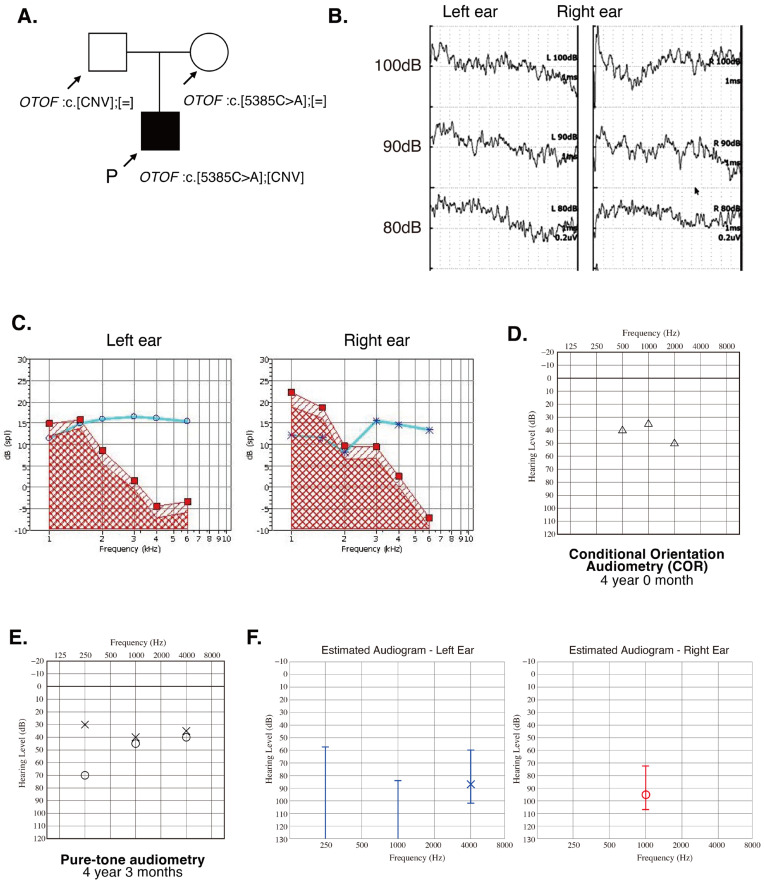
Pedigree and detailed audiometric test results for the patient. (**A**) Pedigree and identified variant. (**B**) ABR test results showed no response even at 100 dB. (**C**) Results of DPOAE showed good response in both ears. (**D**) Hearing thresholds for COR audiometry at 4 y. o. (**E**) Pure-tone audiometry results at 4 years and 3 months of age. (**F**) ASSR thresholds also showed severe-to-profound HL.

The patient was a four-year-old boy with auditory neuropathy spectrum disorder. He was born at 38 weeks of gestation, weighing 3104 g, without any significant events during the perinatal period. He passed the newborn hearing screening test, performed with OAE, and had no family history of HL (Figure 1A). Due to his limited number of meaningful words, the patient visited a local pediatric clinic at 30 months. While the patient’s speech was unclear, some meaningful words were emerging. At 36 months of age, the number of meaningful words had not increased, and he demonstrated some difficulty in understanding instructions. To evaluate potential HL, the patient visited a local ENT clinic. The tympanic membranes and MRI findings were normal. However, the ABR showed no response at 100 dB in either ear (Figure 1B), and the patient was referred to our department at 45 months of age. Distortion product otoacoustic emission (DPOAE) responses were normal (Figure 1C), and the pure-tone audiometry revealed low-frequency HL in the right ear (Figure 1D,E). However, the patient’s ASSR indicated severe HL (Figure 1F). Based on the discrepancy between the DPOAE and ABR/ASSR results, the patient was diagnosed with ANSD.

### 3.2. Short-Read NGS Analysis

To identify the genetic cause of HL in this patient, short-read NGS analysis for 63 target genes, which have previously been reported as causative for non-syndromic HL and syndromic HL, was performed. As a result of the short-read NGS analysis, a novel single nucleotide variant (SNV) in the *OTOF* gene (*OTOF*: NM_001287489:exon43:c.5385C>A:p.F1795L) was identified. The identified SNV was not observed in normal control databases, including gnomAD ver.4.1 [29] and ToMMo60KJPN [30]. In silico prediction scores for the identified SNV were relatively high and supported its pathologic effect on protein function (SIFT: Deleterious, PolyPhen2: Benign, Mutation Taster: Disease causing, Mutation Assessor: Medium, REVEL score: 0.847, CADD Phred score: 25) [31]. This variant is located in the C_2_F domain (one of the multi C_2_-domains of the otoferlin Ca^2+^-dependent membrane-targeting module), which has a crucial role for otoferlin function in vesicular exocytosis [32,33]. This variant (p.F1795L) was novel; however, a different missense variant affecting the same amino acid residue (p.F1795C) has been reported as a pathogenic variant in previous reports [8,34]. The identified variant was classified as “Likely pathogenic” based on the ACMG guidelines (PM1, PM2, PM5, and PP3). We also performed Sanger sequencing analysis for his parents, and found that the proband’s mother carried this variant in a heterozygous state.

**Figure 2:**
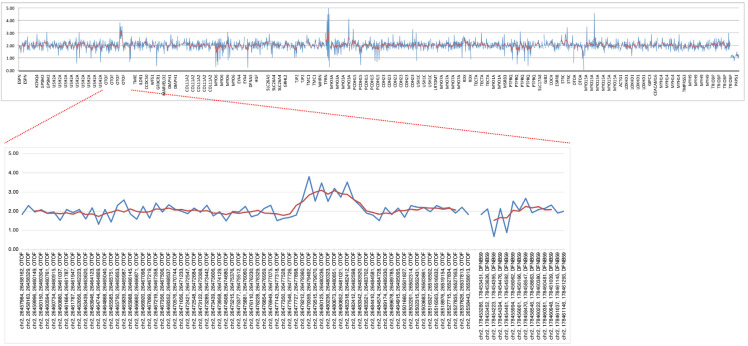
Copy number analysis results using short-read NGS read depth data. The upper panel indicates the all-target genes’ copy numbers and the lower panel indicates the target genes’ copy numbers on chr 2. Blue line indicates the estimated copy number for each amplicon and red line indicates the smoothing value for five relative amplicons.

We then performed CNV analysis using the read depth data obtained from the short-read NGS results according to our previous CNV visualization method [24]. As a result of the CNV analysis, the patient showed one copy gain in the *OTOF* gene (ranging from chr2: 26,479,212 to 26,482,598 of GRCh38.p5), which included the region from exon14 to exon18 (Figure 2). However, our short-read NGS analysis was a target resequencing analysis using super multiplex PCR, so detailed information for the intronic region could not be obtained. In addition, it is impossible to identify more complex changes, including inversion or translocation, based on the short-read NGS data.

### 3.3. Long-Read NGS Analysis

To determine the detailed break point for this patient’s copy number gain, we performed long-read NGS analysis using Oxford Nanopore Technologies PromethION 2 Solo with the adaptive sampling procedure. After long-read NGS, sequence reads were mapped against GRCh38.p5 by using NGMLR, and variant calling and haplotype phasing were performed with Clair3 and WhatsHap.

**Figure 3:**
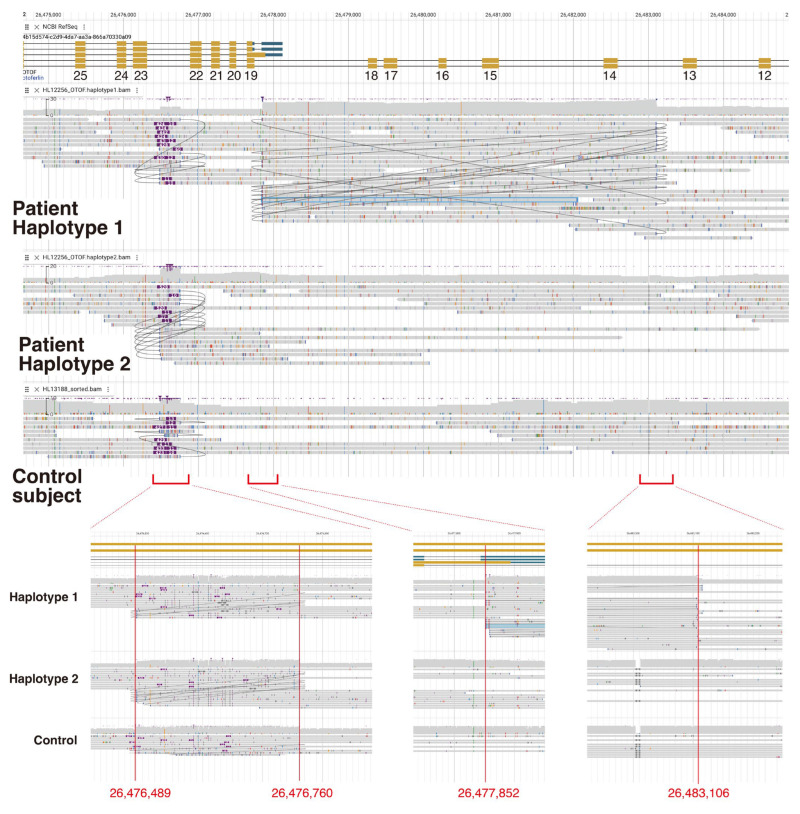
Detailed structural variant analysis of the copy number gain region identified from the proband. The black curved line in the figure shows the line that connects the split sequence reads. **Upper panel:** Read depth distribution and mapped reads onto the region from exon 12 to exon 31 of the *OTOF* gene (NG_009937.1(NM_001287489)). **Lower Left:** High magnification view of the first copy gain region (271-base duplication). Both of the patient’s haplotype-1 and haplotype-2 reads contained duplication of chr2: 26,476,489 to 26,476,760 (located inside intron 22 of the *OTOF* gene NG_009937.1(NM_001287489)). Control subjects also have this duplication. **Lower Middle** and **Right:** High magnification view of the second copy gain region (5254-base duplication). Unlike the first copy gain region, duplication in chr2: 26,477,852 to 26,483,106 (including exon 14 to exon 18 of the *OTOF* gene NG_009937.1(NM_001287489)) was only observed in the patient’s haplotype-1 data.

As a result, we identified two regions of copy number gain caused by the duplication of chr2: 26,476,489 to 26,476,760 (a 271-base duplication located inside intron 22 of the *OTOF* gene NG_009937.1(NM_001287489)) and the duplication of chr2: 26,477,852 to 26,483,106 (a 5254-base duplication including exon 14 to exon 18 of the *OTOF* gene NG_009937.1(NM_001287489)) in haplotype 1 (Figure 3). Further, we identified a duplication of chr2: 26,476,489 to 26,476,760 (271 base duplication) and the *OTOF*: NM_001287489:exon43:c.5385C>A:p.F1795L variant in haplotype 2 (Figure 3).

Interestingly, a copy number gain ranging from 26,476,489 to 26,476,760 was observed in both haplotypes (Figure 3). We therefore estimated this duplication to be a non-pathologic structural variant observed in the Japanese population. We also analyzed the long-read NGS data for some control subjects and confirmed that this CNV was also observed in the controls (Figure 3). Thus, the 271-base duplication between chr2: 26,476,489 and 26,476,760 located in the intronic region is thought to be a non-pathogenic CNV, which is not observed in the reference human genome GRCh38.p5.

Thus, the duplication of chr2: 26,477,852 to 26,483,106 (a 5254-base duplication including exon 14 to exon 18 of the *OTOF* gene NG_009937.1(NM_001287489)) appears to be the candidate causative variant for this patient. As a result of this 5254-base duplication, exon 14 to 18 of the mRNA was duplicated, resulting in a 273-amino acid duplication (p.Gly465_Leu738dup). This amino acid duplication was located in the C_2_C domain (418-to-567 amino acid residues) and resulted in a normal C_2_C domain with duplication of a portion of this domain. Based on our phasing results, this duplication was located in a *trans* configuration with a c.5385C>A variant.

## 4. Discussion

Here, we reported an ANSD patient with a large duplication variant in the *OTOF* gene. To date, 295 variants (including 181 missense or nonsense variants, 46 splicing variants, 62 small insertion/deletion variants, five gross deletion variants and one regulatory region variant) have been reported to be causative for *OTOF*-associated HL or ANSD [35]. However, there are no previous reports of gross insertion or gross duplication variants, and this is the first report of an ANSD patient with a large duplication variant in the *OTOF* gene.

In the ClinVar database, four copy gain variants in the *OTOF* gene have been submitted [36]. The g.(?_26717790)_(26726733_?)dup variant, causing duplication of the region from exon 6–9 of the *OTOF* gene, was also submitted by a single submitter and assigned as “Likely Pathogenic”. The g.(?_26698207)_(26726733_?)dup variant causing duplication of the region from exon 6–25 of the *OTOF* gene was submitted by a single submitter and assigned as “Likely Pathogenic”. The g.(?_26695367)_(26741997_?)dup variant causing duplication of the region from exon 4–30 of the *OTOF* gene was submitted by a single submitter and assigned as “Likely Pathogenic”, and the g.(?_26712059)_(26750808_?)dup variant causing duplication of the region from exon 3–11 of the *OTOF* gene was submitted by a single submitter and assigned as “Likely Pathogenic”. Neither the detailed break points or the direction of duplicated fragments (tandem, inversion, translocation, etc.) were determined for any of the variants. All of these variants were classified as “Likely Pathogenic” based on their large impact on the amino acid sequence. The gross duplication identified in this study (5254-base duplication) was estimated to cause exon 14 to 18 of the mRNA to be duplicated, resulting in a 273-amino acid duplication (p.Gly465_Leu738dup). This amino acid duplication was located in the C_2_C domain (418-to-567 amino acid residues) and led to a normal C_2_C domain with duplication of the portion of this domain. This duplication was located in a *trans* configuration with a p.F1795L variant. Based on the fact that this patient showed a clear ANSD phenotype, we also regarded the gross duplication variant identified in this study as “Likely Pathogenic”.

Oxford Nanopore Technologies adaptive sampling is a powerful tool for increasing the read depth of the target region. Through use of the adaptive sampling procedure, the average read depth of coverage for the target region was increased to 24.5x coverage. The average read depth of coverage for the *OTOF* gene region (chr2: 26,457,203-26,558,756) in the whole genome sequencing results, which were obtained from one PromethION flow cell, was 4.97x coverage (ranging from 3.87 to 6.15). Thus, about five times the data for the target region could be obtained under the same sequencing run conditions. This enables us to perform haplotype phasing analysis to distinguish the phase (*cis* or *trans*) of the identified variants. Indeed, we identified two structural variants (duplication) in this study, and sufficient read depth data allowed us to distinguish the phasing of these two structural variants and SNV (Figure 3).

As for the clinical phenotype of this patient, ABR/ASSR showed profound HL but a good DPOAE response, clearly indicating the ANSD phenotype. Interestingly, this patient showed a discrepancy in hearing thresholds measured by pure-tone audiometry and ABR/ASSR. This may reflect some residual function of the *OTOF* gene, enabling detection of the turning on/off of sound, while making it impossible to follow the continuous stimuli presented in ABR or ASSR testing. Most patients with *OTOF*-associated HL showed congenital or prelingual onset severe-to-profound HL [18]. However, some patients showed atypical clinical phenotypes, such as mild-to-moderate progressive HL [18,37] or temperature sensitive HL [10,38,39]. A recent systematic review of *OTOF*-associated HL also supports these findings [40]. The authors conducted a systematic literature review and analyzed the clinical characteristics of 398 *OTOF*-associated HL patients. Among these 398 patients, 87.2% of the patients showed severe-to-profound HL, whereas 6.8% of patients showed mild-to-moderate HL. Interestingly, among the 154 patients with absent ABR response at the maximum stimulation, 80.5% showed severe-to-profound HL but 9.7% of patients showed mild-to-moderate HL on pure-tone audiometry or behavioral auditory testing. Indeed, some previous reports of *OTOF*-associated HL patients also showed a discrepancy in hearing thresholds, with those measured by pure-tone audiometry or free-field audiometry showing mild-to-moderate HL despite no ABR responses being observed even at 90 dB or 100 dB [41,42,43]. From our results, as well as those previously reported, some portion of *OTOF*-associated HL patients clearly showed mild-to-moderate HL based on pure-tone audiometry but no response on ABR testing. Thus, further study is required to clarify the phenotype divergence and mechanism of this discrepancy in *OTOF*-associated HL.

As a limitation of this study, we could not perform RNA-Seq analysis or a mini-gene plasmid splicing assay to validate the duplication on the mRNA sequences. Further studies are required to determine the effect on mRNA sequences.

## 5. Conclusions

Here, we report the first ANSD patient with a large duplication variant in the *OTOF* gene. We identified a copy number gain by using short-read NGS data and determined the breakpoints by using long-read NGS. The identified variants were novel, but based on the clinical phenotype of the patient, these variants seem to be the genetic cause of this patient’s ANSD. Oxford Nanopore Technologies adaptive sampling is a powerful tool for the analysis of structural variants (particularly for determining the breakpoint and direction) and haplotype phasing.

## Data Availability

The datasets used during the current study are available from the corresponding author on reasonable request.
